# Impact of Bilateral Sympathetic Stellate Ganglionectomy on TGF-β1 Signaling Pathway in Rats With Chronic Volume Overload

**DOI:** 10.3389/fphys.2020.00375

**Published:** 2020-05-14

**Authors:** Mingjing Zhang, Xiaogang Liu, Jie Wu, Yijun Yu, Yuting Wang, Ye Gu

**Affiliations:** Department of Cardiology, Wuhan Fourth Hospital, Puai Hospital, Tongji Medical College, Huazhong University of Science and Technology, Wuhan, China

**Keywords:** transforming growth factor beta 1, bilateral sympathetic stellate ganglionectomy, sympathetic neurohormone, cardiac fibrosis, chronic volume overload

## Abstract

**Background:** We previously reported that bilateral sympathetic stellate ganglionectomy attenuated cardiac remodeling and fibrosis in rats with chronic volume overload. Transforming growth factor beta 1 (TGF-β1) is a polypeptide member of the transforming growth factor beta superfamily of cytokines and actively involved in many pathological processes of cardiovascular diseases. The present study explored the impact of bilateral sympathetic stellate ganglionectomy on the TGF-β1 pathway in this model.

**Methods and Results:** Male Sprague–Dawley rats were randomly divided into sham (S) group, abdominal aorta-cava fistula (AV) group, and bilateral sympathetic stellate ganglionectomy after abdominal aorta-cava fistula (AD) group. Twelve weeks after the abdominal aorta-cava fistula surgery, the myocardial expressions of norepinephrine (NE) and hydroxyproline were significantly higher, while acetylcholine was downregulated in the AV group compared to the S group; the above changes were partly reversed in the AD group. The myocardial expression of TGF-β1 and activity of Smad2/3 phosphorylation were also upregulated in the AV group compared to the S group, which could be reversed by bilateral sympathetic stellate ganglionectomy. *In vitro*, the TGF-β1 expression in cultured myocardial fibroblasts and the proliferation of myocardial fibroblasts were significantly increased post-stimulation with NE in a dose-dependent manner, and these effects could be blunted by co-treatment with a TGF-β1 inhibitor.

**Conclusion:** Our study results indicate that stellate ganglionectomy decreases cardiac norepinephrine release, which leads to decreased TGF-β1 release and reduced fibrosis in rats with chronic volume overload.

## Introduction

Heart failure is a process of progressively pathological disorder, cardiac remodeling and secondary injury after compensatory adaptations, belong to the pathological features of heart failure (Mann and Bristow, 2005). Neurohormone compensation plays an important role in the process of cardiac remodeling, which manifests as sympathetic/parasympathetic imbalance, i.e., an activated sympathetic nervous system accompanied with withdrawal of parasympathetic activity (Packer, 1992; Louridas and Lourida, 2012; Fukuda et al., 2015). Restoring the sympathetic/parasympathetic balance serves as one of the emerging therapy options for heart failure (De Ferrari and Schwartz, 2014; Schrodl et al., 2014; Floras and Ponikowski, 2015). A clinical study of cardiac sympathetic denervation is ongoing for patients of heart failure with reduced ejection fraction (HFrEF), aiming to test whether this strategy could delay the progression of heart failure (Chin et al., 2017). Our previous study found that bilateral sympathetic stellate ganglionectomy (SGX; inferior cervical and T1 ganglia) attenuated cardiac remodeling and fibrosis in rats with chronic volume overload (Zhang et al., 2019). However, the signaling mechanism remains elusive.

Cardiac remodeling is characterized by changes in myocardial mass, size, shape, and accumulation of fibrosis in cardiac ECM in the failing heart (Dhalla et al., 2009). Cardiac fibroblasts are major components of the ECM, which play an important role in cardiac remodeling (Porter and Turner, 2009). Cardiac fibroblasts respond to hormones (such as norepinephrine, NE) and cytokines (such as TGF-β), and the levels of NE and TGF-β are further increased in the myocardial remodeling process. Many of the functional effects of cardiac fibroblasts are mediated through the differentiation of cardiac fibroblasts to myofibroblasts (Yuan et al., 2019). Myofibroblasts play an important role in cardiac ECM remodeling. A previous study showed that a reduction of myofibroblasts could delay the process of cardiac ECM remodeling (Liguori et al., 2019).

Transforming growth factor betas (TGF-βs) are a widely expressed fibrosis regulator factor. TGF-βs include three mammalian isoforms (TGF-β1, TGF-β2, and TGF-β3). The TGF-β subtypes demonstrate sequence homology and have similar mechanisms for processing and activation. TGF-β1 signaling pathways play critical roles in cardiovascular diseases, which might be a potential therapeutic target (Doetschman et al., 2012). TGF-β1 binds to their receptors and related protein and induces cardiac fibroblasts to activate and differentiate into myofibroblasts. Canonical TGF-β signaling promotes cardiac fibrosis by activating Smad2/3 transcription factors through upregulating gene expression (Khalil et al., 2017). The impact of bilateral SGX on the TGF-β1 signaling pathway in the setting of chronic volume overload in a rat model is not fully understood. The aim of this study was therefore to investigate the impact of bilateral SGX on the TGF-β1 signaling pathway and the levels of sympathetic/vagal neurohormones in rats with chronic volume overload.

## Materials and Methods

### Experimental Animal Preparation

Male Sprague–Dawley rats (180–200 g) were subjected to either sham or ACF surgery as described previously in our laboratory (Wu et al., 2015, 2016; Zhang M. J. et al., 2016). “Partial cardiac denervation” was performed by bilateral SGX. That was to say, 7 days after ACF operation, some rats underwent right stellate ganglion and branching resection. After being anesthetized, the rats were intubated endotracheally and connected to an ALCOTT rodent ventilator (model ALC-V8S, China), and then the second rib was cut off on the right to expose the inferior cervical and T1 sympathetic ganglia, known as the stellate ganglion. The stellate ganglion and its nerve branches running into the ganglion were separated from the tissue and cut by micro-forceps. The chest was closed in three separate layers by suturing and a negative intrathoracic pressure was reestablished after surgery. The same group of rats underwent left stellate ganglion and branch resection by the same method after another 7 days (Yoshimoto et al., 2008; Zhang et al., 2019). All procedures were approved and performed according to the Animal Care Committee of Wuhan Fourth Hospital. Animals were kept according to the Guide for the Care and Use of Laboratory Animals published by the United States National Institutes of Health (NIH Publication, 8th edition, 2011). One moribund rat in the aortocaval fistula with bilateral SGX group (AD group) died due to cardiac dysfunction. Data from seven rats in the sham group (S group) and aortocaval fistula group (AV group) and eight rats in the AD group were analyzed.

### Plasma and Myocardial Tissue Biochemistry Examination and Enzyme-Linked Immunosorbent Assay

Twelve weeks after ACF, the rats were sacrificed under deep anesthesia (70 mg/kg sodium pentobarbital, intraperitoneally) after vena cava blood collection. Three rat hearts were randomly selected from each group and longitudinally divided into two portions, then embedded in paraffin for histological examinations. The remaining hearts of each group were divided in their anatomical parts (atria, right and left ventricles with the septum). All samples and tissues were frozen and stored at −80°C for future examinations. The blood was centrifuged at 3,000 × *g* and plasma drawn up into some tubes by pipette for future examinations. Three hundred fifty milligrams of myocardial tissues of the LV and RV, respectively, were placed into a centrifuge tube and added with 700 μl sterile PBS (pH 7.4), the myocardial tissues homogenated, centrifuged at 3,000 × *g*, and the supernatant drawn up by pipette into some tubes for future examinations. Homogenate samples of the plasma, RV, and LV were extracted and acylated using the NE ELISA kit (Abnova, KA1891, Taiwan, China) following the manufacturer’s instructions. NE concentrations of the plasma, RV, and LV were determined with the ELISA kit following the manufacturer’s instructions. The absorbance was recorded at 450 nm. Acetylcholine (ACh) concentrations of the plasma, RV, and LV were determined with the ELISA kit (BioVision, E4454-100, Milpitas, CA, United States) following the manufacturer’s instructions. The absorbance was recorded at 450 nm. We drew up 100 μl plasma into a centrifuge tube and added 200 μl hydrolysate. Twenty micrograms of myocardial tissues of the RV and LV, respectively, was added with 200 μl hydrolysate into a centrifuge tube. Bathed plasma and myocardial tissue with hydrolysate were placed into a centrifuge tube at 95°C for 20 min and the hydrolysis mixture tube mixed once every 10 min to make sure it hydrolyzes sufficiently. The hydroxyproline of the plasma and myocardial tissue was measured following the manufacturer’s instructions (njjcbio, A030-2, Nanjing, China). The absorbance was recorded at 550 nm.

### Quantitation of mRNA Levels

Total RNA was extracted from the LV and RV with the RNeasy Mini Kit (Qiagen, 74104, Hilden, Germany), following the manufacturer’s instructions. PrimeScript RT Master Mix Perfect Real Time (Takara, RR036A, Kusatsu, Shiga, Japan) was used for reverse transcription and cDNA synthesis. Expression of TGF-β1 was detected by QuantiFast SYBR Green PCR Kit (Qiagen, 204057, Hilden, Germany) and Bio-Rad CFX96 (Bio-Rad, C1000, Hercules, CA, United States), following the manufacturer’s instructions. Computation of fold changes in the messenger RNA (mRNA) levels from *C*_T_ values was done using 2^–ΔΔCT^ methods. GAPDH mRNA levels were used as an internal reference standard. Primer sequences are provided in Table 1.

**TABLE 1:** RT-PCR forward/reverse (F/R) primer sequences.

**Forward/Reverse**	**Sequence (5′–3′)**
GAPDH	F: 5′-CGCTAACATCAAATGGGGTG-3′ R: 5′-TTGCTGACAATCTTGAGGGAG-3′
TGF-β1	F: 5′-AAGGAGACGGAATACAGGGCT-3′ R: 5′-ACCTCGACGTTTGGGACTGA-3′

*GAPDH, glyceraldehyde-3-phosphate-dehydrogenase; TGF-β1, transforming growth factor beta 1.*

### Western Blotting

Total proteins were extracted from the LV and RV and protein concentrations were determined through the bicinchoninic acid method. The primary antibodies were as follows: anti-TGF-β1 monoclonal antibody (1:500; Abcam, ab190503, Cambridge, United Kingdom), anti-Smad2/3 monoclonal antibody (1:1,000; Abcam, ab202445, Cambridge, United Kingdom), and anti-pSmad2/3 polyclonal antibody (1:500; Abcam, ab63399, Cambridge, United Kingdom). Secondary antibodies were goat anti-rabbit and goat anti-mouse (KPL, Milford, MA, United States). The images were captured and semi-quantitatively analyzed by the ChemiDoc XRS + System (Bio-Rad, Hercules, CA, United States).

### Cultured Cardiac Fibroblasts *in vitro*, WST-8 Assay, and Cellular Immunofluorescence

Cardiac fibroblasts purchased from iCell Bioscience (RAT-iCell-c002, Shanghai, China) were cultured in PriMed-iCELL-003 (Shanghai, China) supplemented with 10% fetal bovine serum, 10% fibroblast growth factor, 100 U/ml penicillin, and 100 mg/ml streptomycin in a humidified atmosphere of 5% CO_2_ at 37°C. The cells were treated with NE (TargetMol, T7044-1, United States) 0, 5, 10, and 20 μM, respectively, and cultured for 6, 12, and 24 h, respectively, in order to choose the most appropriate concentration and time of NE stimulation.

NE-treated cells were co-cultured with 5 ng/ml TGF-β1 inhibitors (MCE, HY-N0158, China). The cells were seeded at a density of 5 × 10^3^ cells in a 96-well plate and then treated with NE or co-cultured with NE and a TGF-β1 inhibitor to quantify cell proliferation. The water-soluble tetrazolium (WST)-8 assay using a sulfonated tetrazolium salt (Cell Counting Kit-8, Biosharp, China) was performed as recommended in the manufacturer’s instructions. The absorbance was recorded at 450 nm.

Cells were seeded in a 12-well plate with the slides of cells and then treated with NE or co-cultured with NE and a TGF-β1 inhibitor for 12 h. After the supernatant was discarded, the cells were fixed with paraformaldehyde, then treated with Triton X-100, and blocked with serum. After incubation with the first antibody (TGF-β1, 1:200; Abcam, ab190503, United Kingdom) and the second antibody, the cells were then treated with DAPI and observed using a fluorescence microscope.

### Statistical Analysis

Data were shown as the mean ± SD. All data were evaluated for normal distribution using the Shapiro–Wilk test. A non-parametric test was used to analyze non-normal distribution of the data. One-way ANOVA, Tukey’s *post hoc* test, or Games–Howell test was performed to test for differences among the means of various groups. *P* < 0.05 was considered as statistically significant (SPSS Statistics 21.0).

## Results

### Effect of Bilateral SGX on the Expression of Neurohormones in Rats With Chronic Volume Overload

**FIGURE 1:**
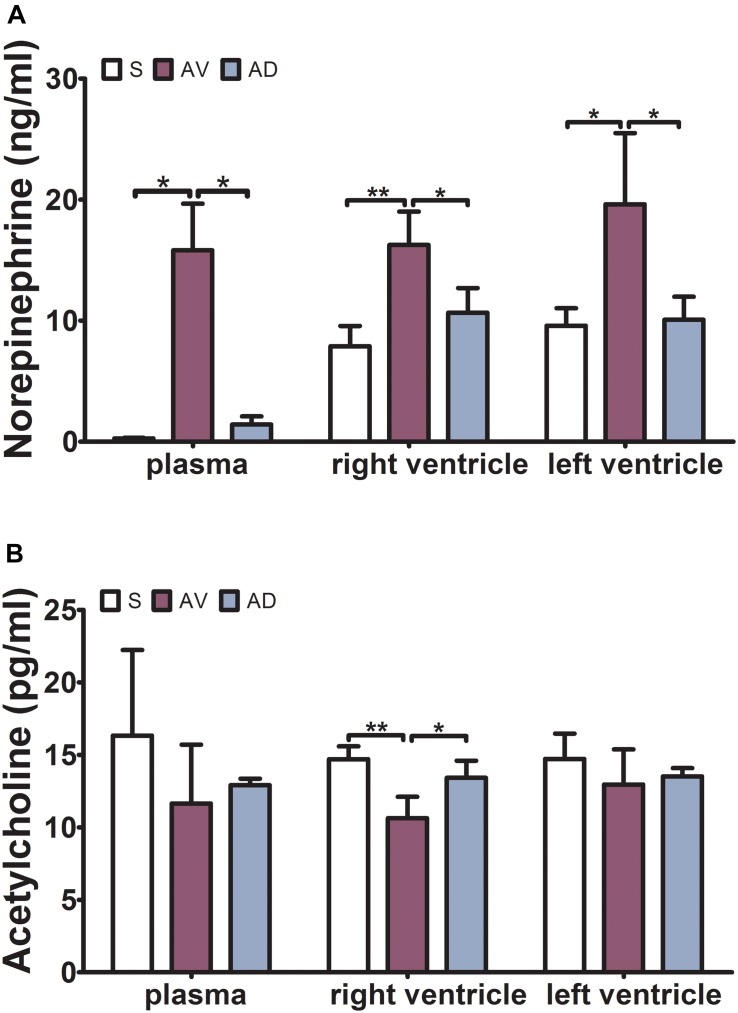
Expressions of norepinephrine (NE) and acetylcholine (ACh) in the plasma, right ventricle (RV), and left ventricle (LV) by ELISA. **(A)** The expression of NE was significantly increased in the abdominal aorta-cava fistula (AV) group and decreased in the bilateral sympathetic stellate ganglionectomy after abdominal aorta-cava fistula (AD) group in the plasma, RV, and LV. **(B)** The expression of ACh was significantly decreased in the AV group and increased in the AD group in the RV (*P < 0.05, **P < 0.01) (three replicate experiments were performed).

The expression of NE was significantly upregulated in the AV group compared to the S group in the plasma, RV, and LV (*P* < 0.05, *P* < 0.01, and *P* < 0.05, respectively) and downregulated in the AD group compared to the AV group in both RV and LV (*P* < 0.05). The expression of ACh was significantly downregulated in the AV group (*P* < 0.01) and upregulated in the AD group (*P* < 0.05) in the RV (Figure 1).

### Impact of Bilateral SGX on Hydroxyproline and TGF-β1 Expression

**FIGURE 2:**
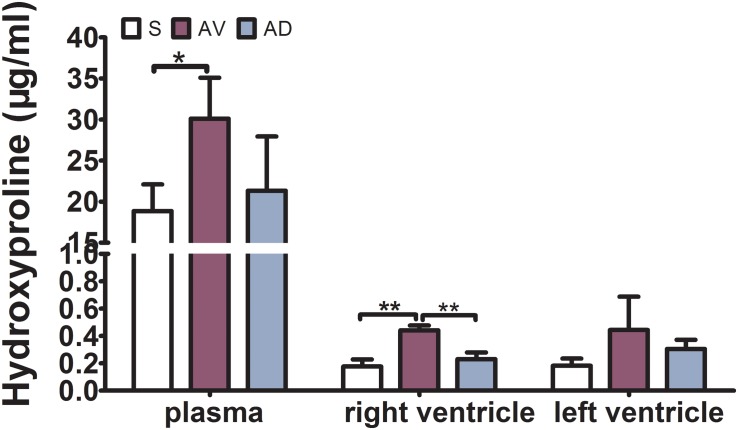
Expression of hydroxyproline in the plasma, RV, and LV by ELISA. The expression of hydroxyproline was significantly increased in the abdominal aorta-cava fistula (AV) group in the plasma and RV (*P < 0.05, **P < 0.01) and significantly reduced in the bilateral sympathetic stellate ganglionectomy after abdominal aorta-cava fistula (AD) group in the RV (**P < 0.01) (three replicate experiments were performed).

**FIGURE 3:**
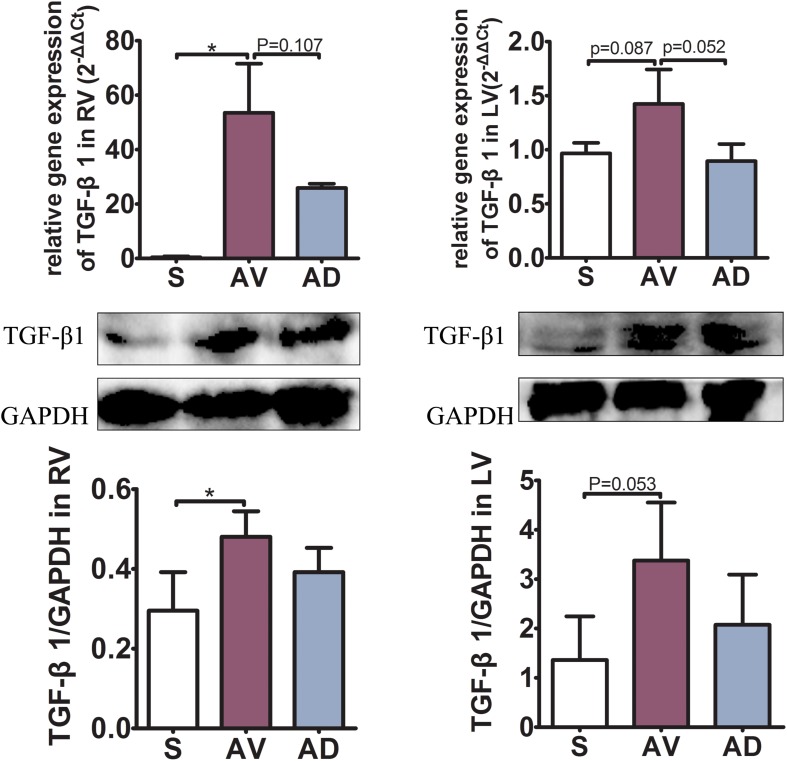
mRNA and protein expressions of TGF-β1 of the myocardial tissue in the right (RV) and left (LV) ventricles. The mRNA and protein expression of TGF-β1 in the RV were significantly increased in the abdominal aorta-cava fistula (AV) group compared to the sham (S) group (*P* < 0.05) and tended to be higher in the LV (*P* = 0.087). The mRNA expression of TGF-β1 tended to be downregulated in the bilateral sympathetic stellate ganglionectomy after abdominal aorta-cava fistula (AD) group compared to the AV group in both the RV and LV (*P* = 0.107 and *P* = 0.052, respectively) (three replicate experiments were performed). *P < 0.05.

The expression of hydroxyproline was significantly increased in the AV group compared to the S group in the plasma and RV (*P* < 0.05 and *P* < 0.01, respectively) and tended to be increased in the LV. It was significantly reduced in the AD group compared to the AV group in the RV (*P* < 0.01) and tended to be reduced in the plasma and LV (Figure 2). The mRNA and protein expression of TGF-β1 in the RV were significantly increased in the AV group compared to the S group (*P* < 0.05) and tended to be higher in the LV (*P* = 0.087). The mRNA expression of TGF-β1 tended to be downregulated in the AD group compared to the AV group in both the RV and LV (*P* = 0.107 and *P* = 0.052, respectively) (Figure 3).

### Myocardial Protein Expression of Smad2/3 Phosphorylation

**FIGURE 4:**
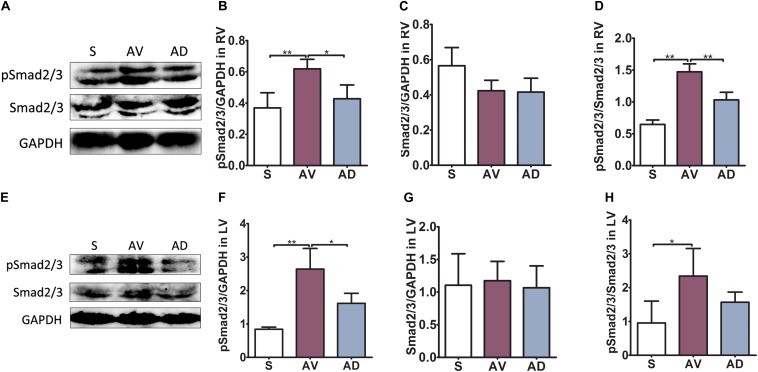
Protein expression of Smad2/3 phosphorylation in cardiac extracellular matrix (ECM) remodeling. In the RV **(A–D)**, the protein expression of pSmad2/3 and the ratio of pSmad2/3 to Smad2/3 were significantly upregulated in the abdominal aorta-cava fistula (AV) group compared to the sham (S) group (both *P* < 0.01) and significantly downregulated in the bilateral sympathetic stellate ganglionectomy after abdominal aorta-cava fistula (AD) group compared to the AV group (*P* < 0.05 and *P* < 0.01, respectively). The protein expression of Smad2/3 was similar among groups in the RV. In the LV **(E–H)**, the protein expression of pSmad2/3 and the ratio of pSmad2/3 to Smad2/3 were significantly upregulated in the AV group (*P* < 0.01 and *P* < 0.05, respectively) and pSmad2/3 was significantly reduced in the AD group (*P* < 0.05). The protein expression of Smad2/3 in the LV was similar among groups (six replicate experiments were performed). *P < 0.05, **P < 0.01.

In the RV, the protein expression of pSmad2/3 and the ratio of pSmad2/3 to Smad2/3 were significantly upregulated in the AV group compared to the S group (both *P* < 0.01) and significantly downregulated in the AD group compared to the AV group (*P* < 0.05 and *P* < 0.01, respectively). The protein expression of Smad2/3 was similar among groups in the RV. In the LV, the protein expression of pSmad2/3 and the ratio of pSmad2/3 to Smad2/3 was significantly upregulated in the AV group (*P* < 0.01 and *P* < 0.05, respectively). pSmad2/3 was significantly reduced in the AD group (*P* < 0.05). The protein expression of Smad2/3 in the LV was similar among groups (Figure 4).

### Proliferation of Cardiac Fibroblasts and Expression of TGF-β1 *in vitro*

**FIGURE 5:**
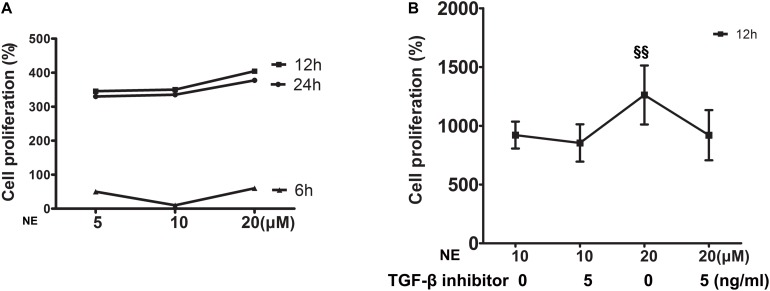
Proliferation of cardiac fibroblasts *in vitro*. **(A)** Cardiac fibroblasts were stimulated with different concentrations of norepinephrine (NE) for 6, 12, and 24 h. The strongest cell proliferation was induced by 20 μM NE at 12 h (four replicate experiments were performed). **(B)** The proliferation of cardiac fibroblasts was higher with 20 μM NE than that of 10 μM NE at 12 h (*P* < 0.01), and this effect could be blunted by co-treatment with a TGF-β1 inhibitor. (^§§^ *P* < 0.01, NE 10 μM vs. 20 μM) (four replicate experiments were performed).

*In vitro*, cardiac fibroblasts were stimulated with different concentrations of NE at various time intervals and the cell proliferation capacity was detected by the WST-8 assay. The results showed that the strongest cell proliferation was induced by 20 μM NE at 12 h (Figure 5A). This effect could be blunted by co-treatment with a TGF-β1 inhibitor (Figure 5B).

**FIGURE 6:**
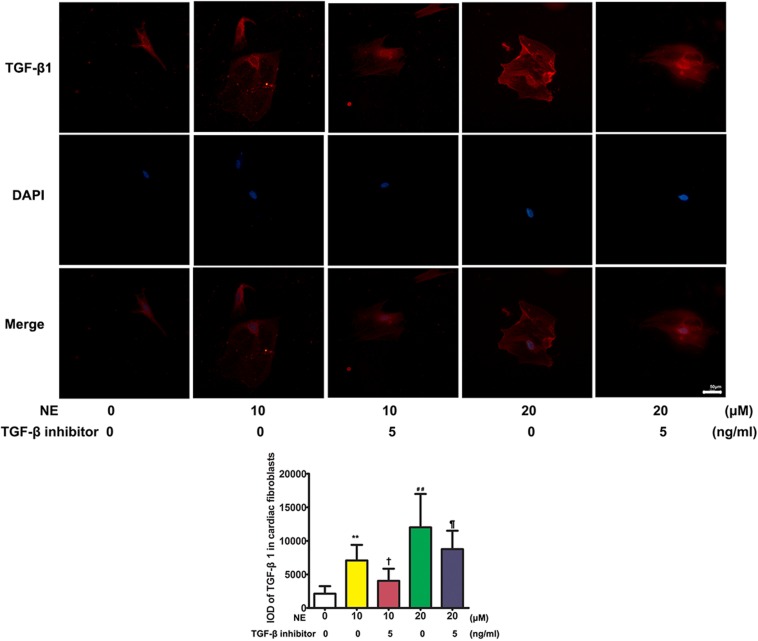
Expression of TGF-β1 in cardiac fibroblasts *in vitro*. The integrated option density (IOD) of TGF-β1 was significantly higher in the norepinephrine (NE) 20 μM group than in the NE 10 μM group (*P* < 0.01), and the IOD of TGF-β1 was reduced after co-treatment with a TGF-β1 inhibitor (*P* < 0.05) *in vitro* (NE 0 vs. 10 μM: **P < 0.01; NE 0 vs. 20 μM: ^##^*P* < 0.01; NE 10 μM vs. NE 10 μM + TGF-β1 inhibitor: ^†^*P* < 0.05; NE 10 μM + TGF-β1 inhibitor vs. NE 20 μM + TGF-β1 inhibitor: ^¶^ *P* < 0.05) (four replicate experiments were performed).

The immunofluorescence results showed that TGF-β1 was significantly upregulated after stimulation with NE in cardiac fibroblasts *in vitro*. The integrated option density (IOD) of TGF-β1 was significantly higher in the NE 20 μM group than in the NE 10 μM group (*P* < 0.01), and the IOD of TGF-β1 was reduced after co-treatment with a TGF-β1 inhibitor (*P* < 0.05) (Figure 6).

## Discussion

**FIGURE 7:**
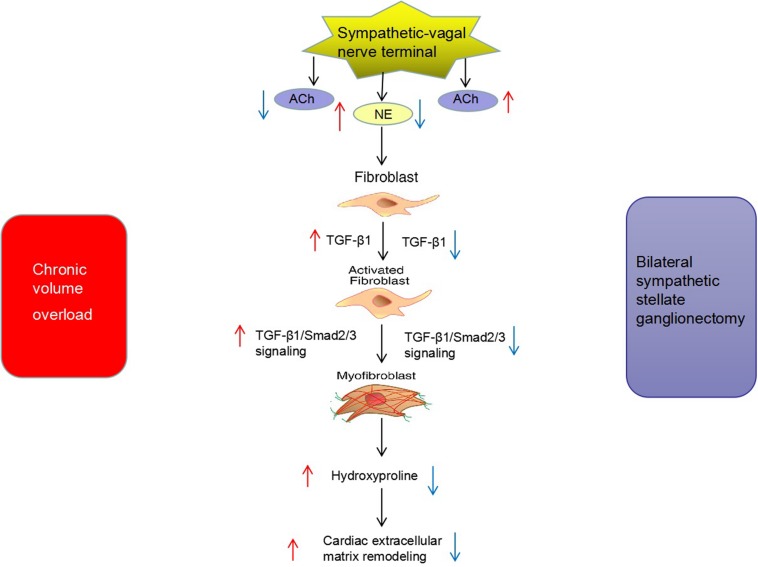
Potential mechanism of bilateral sympathetic stellate ganglionectomy (SGX) on cardiac ECM remodeling in rats with chronic volume overload.

The major findings of the present study were as follows. (1) The secretion of sympathetic neurohormone was increased and vagal neurohormone was decreased in the hearts of rats with chronic volume overload, indicating sympathetic activation in this model. TGF-β1 signaling was also activated in this model. (2) The above changes could be partly reversed by bilateral SGX, suggesting that the previously observed beneficial effects of bilateral SGX in this model (Zhang et al., 2019) were at least partly mediated by downregulating TGF-β1 signaling. (3) *In vitro* results further evidenced the positive causal relationship among NE, TGF-β1, and myocardial fibroblast proliferation. To the best of our knowledge, this is the first report on the impact of bilateral SGX on the TGF-β1 signaling pathway in rats with chronic volume overload (Figure 7).

### Effect of Bilateral SGX on Cardiac ECM Remodeling-Related Signaling Under Chronic Volume Overload

The level of sympathetic neurohormone was closely related to ECM. Briest et al. (2003) showed that NE injection resulted in ECM remodeling in the LV. A previous study also found that sympathetic nerve activity was increased in rats post-myocardial infarction and that dexamethasone could reduce sympathetic innervation and myocardial reactive fibrosis in the myocardial infarction area (El-Helou et al., 2008). We previously showed that bilateral SGX attenuated cardiac remodeling and fibrosis under chronic volume overload. Our previous results showed that the weights of the left and right ventricles and the heart-to-body ratio were increased under chronic volume overload, which were decreased by bilateral stellate ganglionectomy (Zhang et al., 2019). The collagen of heart tissue sections was detected by Sirius red staining. The results showed that the collagen deposition was obviously increased under chronic volume overload and decreased after bilateral stellate ganglionectomy (Zhang et al., 2019). The efferent sympathetic nerve that controls the heart includes the superior cervical ganglia, middle cervical ganglia, stellate ganglion (inferior cervical ganglia and the T1 ganglion fusion), and thoracic sympathetic ganglion II to IV, known as the cardiac sympathetic nerve (Schwartz, 2014). To our surprise, bilateral stellate ganglion denervation resulted in a large decrease in the plasma norepinephrine levels compared to the levels measured in normal rats in this study. The potential reason might be that bilateral stellate ganglion resection induced not only the decrease of norepinephrine secretion but also the decrease of sympathetic activity due to the influence on other vascular beds (such as kidneys) in the setting of volume expansion. The results of the present study evidenced the sympathetic activation in this model, in which sympathetic neurohormone was upregulated and vagal neurohormone was downregulated. These changes were joined by increased hydroxyproline, a characteristic component of collagen (Meng et al., 2019), while bilateral SGX could partly reverse the above changes. Our results thus hinted that the direct anti-sympathetic effects of bilateral SGX could lead to a reduced ECM remodeling. The sympathetic nerve and vagus nerve in the autonomic nerve are a pair of equilibrium bodies. Cerati et al. showed that left stellectomy is accompanied by a reflex increase in cardiac vagal efferent nerve activity and pulse synchronous activity (Cerati and Schwartz, 1991). Li et al. demonstrated that increased vagal activity with chronic electrical stimulation of the vagus nerve could reduce the expression of NE and attenuate cardiac remodeling in heart failure rats (Li et al., 2004). Taken together, strategies aiming to upregulate vagal activity and/or downregulate sympathetic tone, as in the case of bilateral SGX, could effectively reduce cardiac ECM remodeling.

### Impact of Bilateral SGX on TGF-β1 Signaling Pathways in the Setting of Chronic Volume Overload

Transforming growth factor betas were a multifunctional cytokine secreted by a variety of cells with multiple physiological and pathological functions (Doetschman et al., 2012). The close relationship between TGF-β activity and sympathetic activity serves as a focus in recent research. Vega et al. (2009) showed that superior cervical ganglionectomy (SCGX) could downregulate TGF-β and reduce ocular sympathetic nerve activity. TGF-β receptors are widely distributed on the membrane of myofibroblasts and could bind to TGF-β, mediating the downstream signaling pathways involved in cardiac ECM remodeling and fibrosis (Fix et al., 2019). Smad2/3 is one of the signaling pathways regulated by TGF-β1, and TGF-β1 could mediate the phosphorylated activity of Smad2/3 and participate in ECM remodeling (Zhang et al., 2016; Han et al., 2018). Our study demonstrated the upregulated expression of TGF-β1 and Smad2/3 phosphorylation in this chronic volume overload model. It is known that activated Smad2/3 phosphorylation signaling pathway could promote collagen synthesis (Khalil et al., 2017). After bilateral SGX, the expression levels of both TGF-β1 and phosphorylated Smad2/3 were significantly downregulated in this chronic volume overload model. The above results suggested that the beneficial effects of bilateral SGX in this model are partly mediated by downregulating the upregulated TGF-β1 expression and Smad2/3 phosphorylation.

### Changes of Cardiac Fibroblasts Stimulated by NE

To verify the causal relationship between NE stimulation and TGF-β1 signaling, we stimulated the cardiac fibroblasts *in vitro* with NE of various concentrations at different time intervals. The results showed that the proliferation capacity of cardiac fibroblasts and TGF-β1 expression in these cells were increased after being cultured with NE, while the TGF-β1 inhibitor could blunt the above changes *in vitro*, indicating the causal relationship between NE, TGF-β1, and cardiac fibroblast proliferation. A previous study by Akiyama-Uchida et al. (2002) demonstrated that NE enhanced cardiac fibrosis through TGF-β1 post-receptor signaling, predominantly *via* the p38 MAP kinase pathway. NE enhances fibrosis mediated by TGF-β in cardiac fibroblasts (Akiyama-Uchida et al., 2002).

### Study Limitations

There were some study limitations in this study. Firstly, bilateral sympathetic stellate ganglionectomy was not performed in sham animals as the control group because the members of the Ethics Committee of our hospital did not approve this operation in normal animals. They argued that this adds extra harm to animals without clinical significance. Therefore, we had to focus on the impact of bilateral sympathetic stellate ganglionectomy on the TGF-β1 signaling pathway of rats with volume overload. Secondly, it is important to measure the hemodynamics data to show the effects of stellate ganglionectomy. Future study is warranted to clarify this issue. Thirdly, TGF-β1 knockout rats were not used due to technical limitations. The expression of TGF-β1 was inhibited only by the chemical reagent in this study. Future study is warranted to use genetic methods such as miRNA, siRNA, etc., to inhibit TGF-β1 gene expression and protein synthesis to define the related mechanisms.

## Conclusion

In conclusion, our study indicates that the activation of the sympathetic nervous system is one of the mechanisms in a failing heart induced by chronic volume overload. Bilateral SGX could reduce the expression of sympathetic neurohormone. This effect is partly mediated by downregulating TGF-β1 as well as Smad2/3 phosphorylation in rats with chronic volume overload. Our findings provide experimental and molecular evidence for the treatment of heart failure by bilateral SGX.

## Data Availability Statement

The datasets generated for this study are available on request to the corresponding author.

## Ethics Statement

This study was carried out in accordance with the principles of the Guide for the Care and Use of Laboratory Animals of the United States National Institutes of Health (NIH Publication, 8th Edition, 2011). The protocol was approved by the Animal Care Committee of Wuhan Fourth Hospital.

## Author Contributions

MZ and YG contributed to the conception and design of research. MZ and YY analyzed the data. MZ and XL interpreted the results of experiments. MZ drafted the manuscript. YG edited and revised the manuscript. MZ, XL, and JW performed the experiments. All authors approved the submitted version of the manuscript.

## Conflict of Interest

The authors declare that the research was conducted in the absence of any commercial or financial relationships that could be construed as a potential conflict of interest.

## Abbreviations

ACF: abdominal aorta-cava fistula
ACh: acetylcholine
ECM: extracellular matrix
IPP: Image ProPlus
LV: left ventricle
NE: norepinephrine
RV: right ventricle
SGX: sympathetic stellate ganglionectomy
TGF- β 1: transforming growth factor beta 1.
